# 7,8-Dihydroxyflavone Ameliorates Cognitive Impairment by Inhibiting Expression of Tau Pathology in ApoE-Knockout Mice

**DOI:** 10.3389/fnagi.2016.00287

**Published:** 2016-11-29

**Authors:** Yang Tan, Shuke Nie, Wende Zhu, Fang Liu, Hailong Guo, Jiewen Chu, Xue B. Cao, Xingjun Jiang, Yunjian Zhang, Yuzhen Li

**Affiliations:** ^1^Department of Neurology, Union Hospital, Tongji Medical College, Huazhong University of Science and TechnologyWuhan, China; ^2^Department of Neurology, Renmin Hospital of Wuhan UniversityWuhan, China; ^3^Department of Neurosurgery, Union Hospital, Tongji Medical College, Huazhong University of Science and TechnologyWuhan, China; ^4^Department of Medicine, LuoHu Chronic Disease Control and Cure HospitalShenzhen, China; ^5^Department of Pharmacy, The Eighth Affiliated Hospital of Sun Yat-sen UniversityShenzhen, China

**Keywords:** 7, 8-dihydroxyflavone, ApoE knockout, cognitive impairment, neuroprotective, tau pathology, asparaginyl endopeptidase, protein kinase B, glycogen synthase kinase-3β

## Abstract

7,8-Dihydroxyflavone (7,8-DHF), a tyrosine kinase B agonist that mimics the neuroprotective properties of brain-derived neurotrophic factor, which can not efficiently deliver into the brain, has been reported to be useful in ameliorating cognitive impairment in many diseases. Researches have indicated that apolipoprotein E-knockout (ApoE-KO) mouse was associated with cognitive alteration via various mechanisms. Our present study investigated the possible mechanisms of cognitive impairment of ApoE-KO mouse fed with western type diet and the protective effects of 7,8-DHF in improving spatial learning and memory in ApoE-KO mouse. Five-weeks-old ApoE-KO mice and C57BL/6 mice were chronically treated with 7,8-DHF (with a dosage of 5 mg/kg) or vehicles orally for 25 weeks, and then subjected to Morris water maze at the age of 30 weeks to evaluate the cognitive performances. Afterward, histology analysis and western blotting were performed. Spatial learning and memory deficits were observed in ApoE-KO mice, which were consistent with higher expression of active-asparaginyl endopeptidase (active-AEP) as well as AEP-derived truncated tau N368 compared with normal group. In addition to that, long-term treatment of 7,8-DHF dramatically ameliorated cognitive decline in ApoE-KO mice, accompanied by the activation in phosphorylated protein kinase B (Akt)/glycogen synthase kinase-3β (GSK-3β) pathway and down-regulated expression of tau S396 and PHF-tau (phosphorylated tau at ser396 and ser404 epitope). These findings suggested that cognitive impairment of ApoE-KO mouse might associate with tau pathology and 7,8-DHF could activate AKT and then phosphorylate its downstream molecule to inhibit expression of abnormal tau, meanwhile, 7,8-DHF could reduce the expression of active-AEP and then inhibit production of truncated tau N368.

## Introduction

Apolipoprotein E (ApoE) has been closely linked to AD-associated pathology, like neurofibrillary tangles and amyloid plaques. Scientists have reported cognitive impairment in ApoE knockout (ApoE-KO) mice (Liu et al., 2013), with cholinergic deficits (Gordon et al., 1995), decreased synaptic excitability (Veinbergs et al., 1999), amyloid-β (Aβ) deposition, and impaired function of synaptosomes (Keller et al., 2000). ApoE-KO mouse fed with high cholesterol diet would result in severe atherosclerosis, leakage of blood-brain barrier, neuronal apoptosis and cognitive impairment (Bink et al., 2013). Furthermore, scientists have proved that loss of ApoE would lead to tau phosphorylation increase (Genis et al., 1995; Bi et al., 2001). Whether tau pathology, like truncated tau and phosphorylated tau, could aggravate cognitive deficits of ApoE-KO mouse fed with western type diet was still unknown.

Brain-derived neurotrophic factor (BDNF), as a member of neurotropic family in nervous system, has various therapeutic effects via activating tyrosine kinase (TrkB). However, poor delivery and short half-life *in vivo* hamper its clinical trials (Pardridge, 2007). 7,8-dihydroxyflavone (7,8-DHF) is the first promising small molecular TrkB agonist which fully mimics the physiological properties of BDNF (Liu et al., 2016). As a bioavailable chemical, 7,8-DHF can penetrate brain blood barrier upon intraperitoneal or oral administration to provoke TrkB and its downstream signaling, such as phosphoinositide 3-kinase/protein kinase B (Akt; Wu et al., 2014), to exert its central role in pathologic conditions. As a consequence of activating TrkB, 7,8-DHF inhibits obesity in female mice (Chan et al., 2015), reduces spine morphology abnormalities in fragile X syndrome (Tian et al., 2015), enhances axon regeneration and muscle renovation after peripheral nerves injuries (English et al., 2013), improves motor function and prolongs survival in Huntington’s disease (Jiang et al., 2013). Furthermore, it can reverse synapse loss and prevent memory deficits in Alzheimer’s disease (AD; Zhang et al., 2014a; Gao et al., 2016).

Glycogen synthase kinase-3β (GSK-3β) is a proline-directed serine/threonine protein kinase. Its kinases activity can be inhibited by the phosphorylation of Ser9 through BDNF/TrkB/AKT pathway. As one of the major tau kinases, expression of GSK-3β leads to tau hyperphosphorylation at many sites *in vitro* (Rankin et al., 2007) and *in vivo* (Liu et al., 2003), like ser396 and ser404, the favorite sites for GSK-3β (Liu et al., 2007). With phosphorylation at ser396 and ser404 by GSK3β, the pathological conformation of tau becomes tighter and enhances the aggregation to PHF-tau (phosphorylated tau at ser396 and ser404 epitope). The abnormal tau could be recognized by monoclonal antibody PHF1 (Jeganathan et al., 2008). Due to its attractive candidate for modulating AD-like pathology, researchers have found several small-molecule inhibitors of GSK-3β to reduce tau pathology, especially tau S396, *in vivo* (Selenica et al., 2007). In addition to that, several articles have demonstrated that by activating AKT, the expression of p-GSK3β (Ser9) was up-regulated, following with decreased expression of tau S396 (Jiang et al., 2015; Ren et al., 2015). Furthermore, scientists have also proved that inhibition of AKT could result the increased activity of GSK3β correlated with higher expression of tau S396 and cognitive impairment (Liu et al., 2003). Thus, we supposed that 7,8-DHF could promote phosphorylation of GSK3β via AKT/GSK pathway and suppress activation of GSK-3β to diminish tau Ser396 and PHF1.

Besides hyperphosphorylated tau, Zhang et al. (2014b) recently showed that the enzyme asparagine endopeptidase (AEP) cleaved tau at residues 255 and 368 directly. However, the tau fragment, derived by active-AEP, that does display most of the toxic effects is tau 1-368. These fragments are toxic independently of phosphorylation state (Zhang et al., 2014b). AEP, a lysosomal cystein proteinase, is activated in low PH circumstances and cleaves protein substrates on the C-terminal side of asparagine (Rosenmann, 2014). We wonder if levels of active-AEP would increase in ApoE-KO mice with severe atherosclerosis, and whether 7,8-DHF could inhibit its pathological influence or not.

Studies have proved that 37-weeks-old male ApoE-KO mouse fed with low-fat diet did not shown evidence of AD-like pathology such as amyloid plaques and PHF (Moghadasian et al., 2001). In our study, male ApoE-knockout mice and C57BL/6 wild-type mice were fed with western type diet with high levels of cholesterol for 25 weeks and Morris water maze (MWM) tests were performed at the age of 30 weeks to evaluate behavioral performances.

In conclusion, in the present study, we investigated 7,8-DHF potential neuroprotective effects in ApoE-KO mouse by evaluating the behavioral performances and histological analysis of hippocampus and cortex. We hypothesized that 7,8-DHF could alleviate cognitive deficits via activating TrkB and its downstream signals as well as suppressing activation of AEP. We also hypothesized that, comparing with C57BL/6 mouse, ApoE-KO mouse, with severe cerebrovascular atherosclerosis, expressed more tau pathology, which could aggravate cognitive impairment.

## Materials and Methods

### Animal

Male ApoE-KO mice and age-matched male C57BL/6 wild-type controls at 4 weeks of age, weighting 16–18 g, were purchased from HFK Bioscience Company (Beijing, China). They were housed under regulated temperature (21–23°C) with 12 h light/dark cycle with food and water available. All the mice were fed separately. This study was implemented conforming to the Rules of Animal Care and Use Committees of Huazhong University of Science and Technology with good laboratory practice and standard operating procedure.

### Materials

7,8-DHF was purchased from TCI company (Tokyo, D1916) and dissolved at 100 mg/ml concentration in 85% saline with 10% dimethylsulfoxide and 5% tweeen-20 (Solarbio, T8220), Akt (pan; C67E7; #4691), phospho-Akt (Ser473; D9E) XP (#4060), phospho-GSK-3β (Ser9; 5B3; #9323) were all rabbit monoclonal antibodies and purchased from Cell Signaling. GSK-3β Rat mAb (#272536), Legumain/Asparaginyl Endopeptidase (AEP) Sheep pAb (AF2058) were purchased from R&D System. Phosphor-Tau (S396) Rabbit mAb (ab109390), PHF1 Rabbit mAb (ab184951), BDNF Rabbit mAb (EP1293; ab108383), TrkB Rabbit pAb (ab33655), and phospho-TrkB Rabbit pAb (Y816; ab75173) were all purchased from Abcam. Tau N368 rabbit antibody was a gift from Prof. Ye of Emory University, USA.

### Cognitive Impairment of ApoE-KO Mouse

After 7 days acclimatization period, all mice were fed with western type diet purchased from HFK Bioscience Company (Beijing, China, H10141) for 25 weeks. All the mice were randomly divided into three groups. The first group was normal group containing 10 C57BL/6 wild-type mice. The second group was cognitive impairment model group with 10 ApoE-KO mice in it. The third group was intervention group in which ApoE-KO mice were treated with 7,8-DHF chronically at the dose of 5 mg/kg (Devi and Ohno, 2012; Zeng et al., 2012; Gao et al., 2016) daily by oral administration for 25 weeks while the mice in other two groups were given vehicles daily. During the experiment, all the mice were measured weight and monitor health status every week. All experimental researches were conducted at the same phase during the day.

### The Morris Water Maze

After intervention for 25 weeks, MWM tests, which is commonly thought to be related with hippocampal-dependent spatial learning and memory (D’Hooge and De Deyn, 2001), were carried on to assess behavioral performance of ApoE-KO mice and protective effects of 7,8-DHF on cognitive decline. The experiment was performed as previously described (Vorhees and Williams, 2006). A 120 cm diameter tank was filled with water with non-toxic milk powder dissolved in it. The pool was divided equally into four quadrants: northeast (NE), southeast (SE), southwest (SW), and northwest (NW). The circular platform was 10 cm in diameter located in the center of NE quadrant, and was submerged 1.5 cm below the surface of water. Several visual cues were placed in the room as spatial reference for mice to locate the invisible platform, and experimenter remained stationary in a fixed location. After 5 days of acquisition trials, the platform was removed and mice were placed in the SW quadrant, opposite to the former platform position and the probe trial underwent. During the whole trials, all mice were maintained on their western type diet and oral administrated by 7,8-DHF or vehicle. Behavioral parameters (latency; percentage of time and distance in NE quadrant; the number of mouse crossing over the position of the platform) were recorded and evaluated with AVTAS software.

All the data were analyzed using SPSS to determine the significance of differences.

### Tissue Preparation

Twenty-four hours after behavioral experiment, six mice of each group were killed by decapitation under anesthesia with isoflurane. Hippocampus and brain cortex were immediately dissected on ice and snap frozen in liquid nitrogen, then stored at -80°C until used. Four mice from each group were transcardially perfused with 4% paraformaldehyde and brains were removed and fixed in 4%PFA at 4°C.

### Western Blotting

Protein preparation was carried out as previously described (Nie et al., 2015). A total of 42 μg protein of each sample were electrophoresed on 10% SDS-PAGE, then the protein samples were transferred to a PVDF membrane (Millipore, USA, NL1253). The membranes were blocked with 5% milk/PBST for 2 h at room temperature and incubated with a variety of primary anti-bodies overnight at 4°C: rabbit monoclonal anti-BDNF (1:1000, Abcam), rabbit polyclonal anti-TrkB (1:1000, Abcam) and rabbit polyclonal anti-phospho-TrkB (Y816; 1:1000, Abcam), rabbit monoclonal anti-Akt (pan; 1:1000), rabbit monoclonal anti-phospho-Akt (Ser473; 1:2000), rabbit monoclonal phospho-GSK-3β (Ser9; 1:1000), GSK-3β Rat mAb (1:2000), Phosphor-Tau (S396) Rabbit mAb (1:4000), PHF1 Rabbit mAb (1:1000), Tau N368 rabbit antibody (1:2000), sheep polyclonal anti-AEP (1:8000). Then, the membranes were washed with TBST for three times (10 mins/times) and incubated with HRP-conjugated secondary anti-rabbit antibody (1:4000 for p-AKT, 1:2000 for others) or anti-sheep antibody (1:2000) for 2 h at room temperature. After washing for three times, the immunoreactive bands were detected by Bio-Rad imaging system and quantified via Image J software.

### Immunofluorescent Staining

Four brain tissues of each group with formaldehyde solution for 24 h were embedded in paraffin, making pathological section. The primary antibody is rabbit antibody tau N368 (1:500) for hippocampus. After all the processes mentioned before (Nie et al., 2015), fluorescent images of hippocampal were examined on inversion fluorescence microscope (NIKON DS-U3). Then the optical density was evaluated via image J software.

### Statistic Analysis

Statistical analysis was performed with SPSS Statistics 22.0 software. The significant differences of data were detected with one- or two-way analysis of variance (ANOVA) followed by LSD. Data were presented as means ± SEM and the significant level was set for *p* < 0.05.

## Result

### 7,8-DHF Ameliorates Cognitive Impairment in ApoE-KO Mice

**FIGURE 1:**
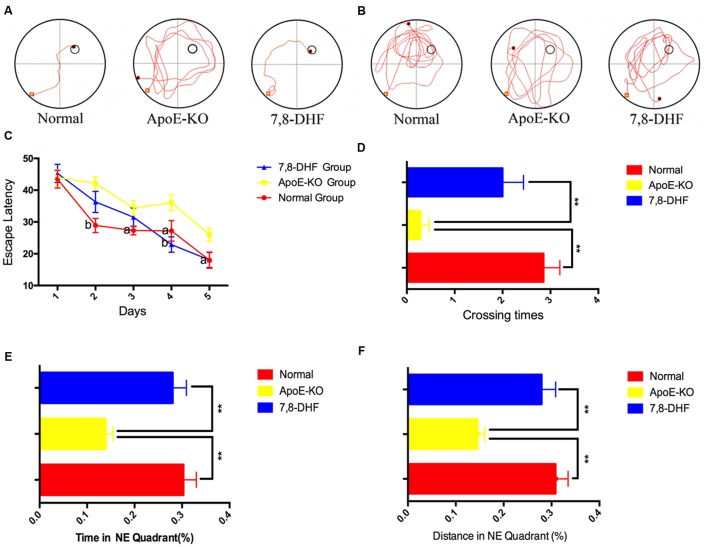
**(A,B)** The swimming trajectory of each group on the last day of training trails **(A)** and probe trail **(B)**; **(C)** The statistical analysis of escape latencies in 5 days training trails; **(D)** Times that the mouse crossed the place where submerged platform was placed were analyzed; In the probe trail, the mouse swimming times **(E)** and distances **(F)** in NE quadrant. Normal, C57BL/6 mouse administrated with vehicles (10%DMSO++5%Tween20+85%saline); ApoE-KO, ApoE-KO mouse administrated with vehicles (10%DMSO+5%Tween20+85%saline); 7,8-DHF, ApoE-KO mouse administrated with 7,8-DHF. Values were presented as mean ± SEM. ^∗∗^*p* < 0.01, ^a^*P* < 0.05 versus the ApoE-KO group;^b^*P* < 0.01 versus the ApoE-KO group.

We performed the MWM to demonstrate whether 7,8-DHF could rescue deficiency of cognition in ApoE-KO mice or not. The swimming trajectories of mouse from each group on the last day of training trails (**Figure 1A**) and probe trail (**Figure 1B**) were shown in **Figure 1**. Measured by two-way ANOVA (day^∗^treatment), the escape latencies of each group during training trails showed remarkable differences and learning was occurring within each group (**Figure 1C**). Compared with normal group, ApoE-KO group performed poorly on training days except for the 1st day (*p* < 0.01 on the 2nd day and *p* < 0.05 on the following days). At the last 2 days of training trails, 7,8-DHF-treated group showed preference for the target hidden platform compared with ApoE-KO mouse (*p* < 0.01 and *p* < 0.05, respectively). No significant difference was found between normal group and 7,8-DHF-treated group in escaping from the pool. After training trails for 5 days, the platform was removed and probe trial testing began. We found out that both the normal group and 7,8-DHF-treated group crossed over the zone where the hidden platform was located more frequent than ApoE-KO group (both *p* < 0.01, **Figure 1D**). Furthermore, compared with ApoE-KO group, larger percentage of swimming distance (**Figure 1E**) and time (**Figure 1F**) in target quadrant were performed in normal group (*p* < 0.01) and 7,8-DHF-treated group (*p* < 0.01). Notably, both normal group and 7,8-DHF-treated group showed similar performances in probe trial testing (*p* > 0.05). Based on the results above, we proved that long-termed treatment with 7,8-DHF could ameliorate the impairment of spatial learning and memory in ApoE-KO group and ApoE-KO mice with western type diet have poorer cognitive level than C57BL/6 mice.

### 7,8-DHF Affects AKT/GSK3β Mediated Pathway in ApoE-KO Mice

**FIGURE 2:**
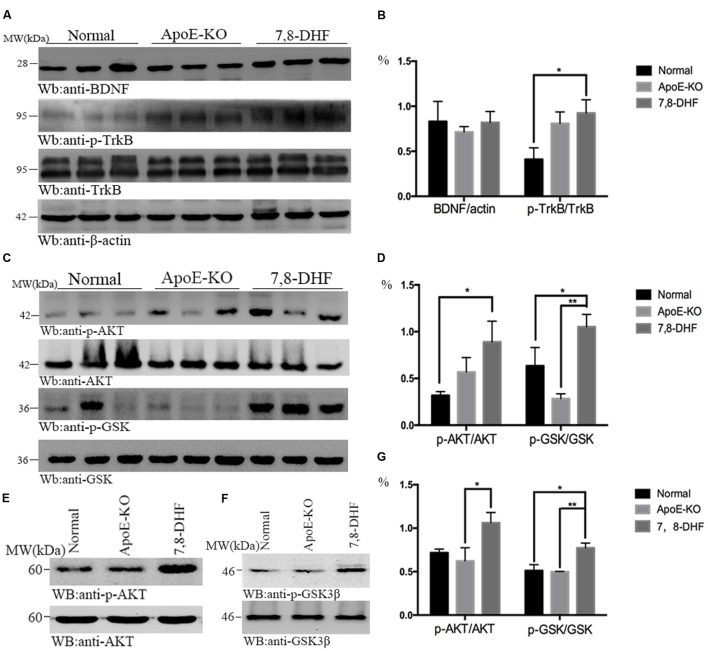
**In hippocampus, the western blotting showed that expression of p-TrkB was more in 7,8-DHF-treated group than in normal group** **(A,B)**, so were the downstream pathways involving AKT/GSK3β **(C,D)**. In cortex, AKT/GSK3β also showed significant differences between each group **(E–G)**. ^∗^*p* < 0.05, ^∗∗^*p* < 0.01. Data were presented as mean ± SEM.

To investigate whether amelioration of cognitive impairment in ApoE-KO mice correlated with 7,8-DHF treatment, we assessed the alteration of BDNF/TrkB/AKT pathway with western blotting (**Figures 2A–D**). As we expected, compared with normal group, chronic long-term administration of 7,8-DHF dramatically up-regulated the expression of TrkB-phosphorylation (p-TrkB) and AKT-phosphorylation (p-AKT) in ApoE-KO mice (both *p* < 0.05) of hippocampus. GSK3β-phosphorylation protein in hippocampus expressed at higher level in the 7,8-DHF-treated group compared with normal group (*p* < 0.05) and ApoE-KO group (*p* < 0.01). All the three groups expressed similarly in BDNF. Meanwhile, western blotting further showed that the expression of p-AKT (*p* < 0.05; **Figures 2E,G**) and p-GSK3β (*p* < 0.01; **Figures 2F,G**) in cortex were up-regulated in 7,8-DHF-treated group compared with ApoE-KO group. Difference was also found between normal group and 7,8-DHF-treated group on expression of p-GSK3β (*p* < 0.05; **Figures 2F,G**). Together, the results suggested that in 7,8-DHF-treated group, significant differences were found in the expression of p-TrkB, p-AKT, and p-GSK3β, which suggested that 7,8-DHF might play a role in ameliorate cognitive impairment in ApoE-KO mice by regulating AKT/GSK3β pathway.

### 7,8-DHF Reduces Tau Pathology in ApoE-KO Mice Via Inhibiting Activation of GSK-3β and AEP

**FIGURE 3:**
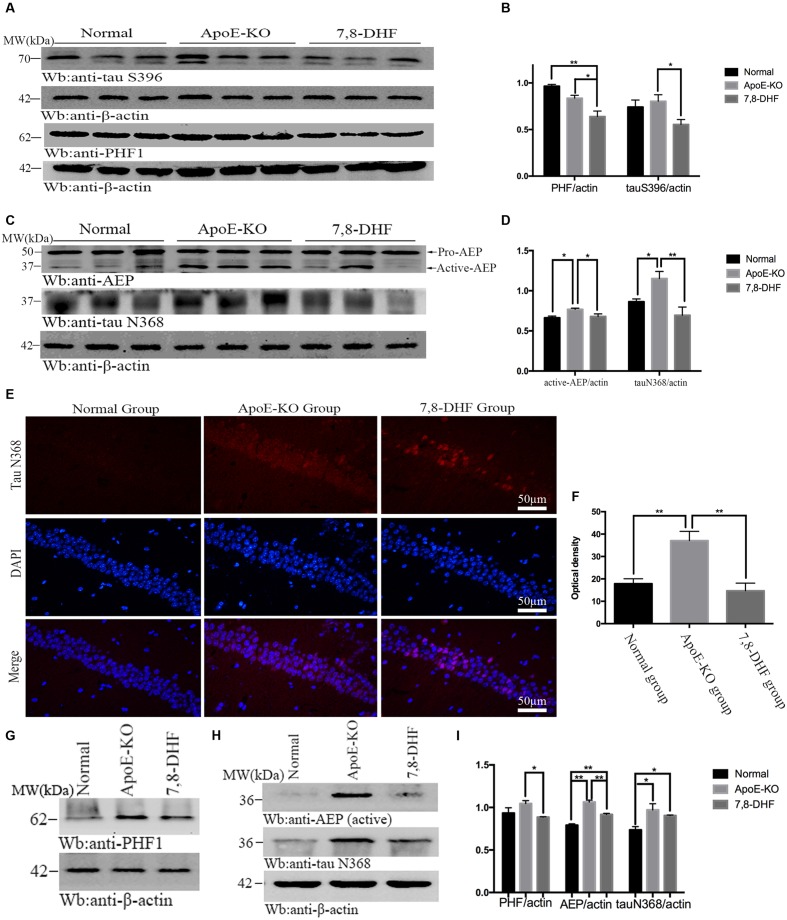
**Western blotting showed expression of tau S396 and PHF1 in hippocampus and the statistical analysis **(A,B)**.** In addition to that, expression of active-AEP and tau N368 of each group in hippocampus were also analysis **(C,D)**. Immunofluorescent staining further reconfirmed the findings that ApoE-KO mouse expressed more tau N368 in hippocampus than C57BL/6 mosue, while 7,8-DHF decrease the expression of tau N368 compared with ApoE-KO mouse **(E,F)**; In cortex, expression of PHF1, active-AEP, and tau N368 were showed by western blotting **(G,H)**, and significant differences were found between groups **(I)**. ^∗^*p* < 0.05, ^∗∗^*p* < 0.01. Data were presented as mean ± SEM. Scale bar = 50 μm.

As mentioned before, 7,8-DHF could inhibit activation of GSK-3β by activating TrkB/AKT mediated pathway. GSK-3β phosphorylates tau at individual sites, especially at S396, as its favorite site. Furthermore, tau phosphorylation is thought to take responsibility for accumulation of paired helical filaments (PHF). So in order to determine the effects of 7,8-DHF on levels of tau pathology of cognitive impairment ApoE-KO mice, we evaluated the alteration of abnormal tau by PHF1 antibody, which can recognize neurofibrillary lesions of brain with higher affinity for phosphorylation of tau at serine residues 396, and tau S396 antibody. We found that, compared with ApoE-KO group (**Figures 3A,B**), 7,8-DHF-treated group dramatically down-regulated the expression of tau S396 (*p* < 0.05) and PHF1 (*p* < 0.05) of hippocampus. In cortex (**Figures 3G,I**), there were also significant differences between ApoE-KO group and 7,8-DHF-treated group in PHF1 expression (*p* < 0.05). In addition to phosphorylated tau, expression of truncated tau N368 was also evaluated. As, we all known the pH in the brain gradually decreases during aging and AEP can be activated in acid circumstances. The active-AEP fragments tau at N368 site that elicits tau hyperphosphorylation and aggregation and provokes neurofibrillary degeneration. But whether the cognitive impairment of ApoE-KO mice with high-fat diet had relation with active-AEP and the following truncated tau and whether 7,8-DHF could decrease tau truncation by inhibiting activation of AEP were still unknown. Indeed, in hippocampus (**Figures 3C,D**), we found significant differences in expression of active-AEP in both 7,8-DHF-treated group (*p* < 0.05) and normal group (*p* < 0.05) compared with ApoE-KO group. Meanwhile, we further showed that expression of tau N368 was higher in ApoE-KO group than normal group (*p* < 0.05) and 7,8-DHF-treatment can dramatically reduced expression of tau N368 compared with ApoE-KO group (*p* < 0.01). In addition to western blotting, we also found similar result in immunofluorescence (**Figures 3E,F**). Brain sections contained the hippocampus regions were stained with tau N368 to assess expression of tau pathology. Tau N368 in hippocampal regions were significantly elevated in the ApoE-KO group compared with normal group (*p* < 0.01) and 7,8-DHF-treated group (*p* < 0.01). But no statistical significance was found between normal group and 7,8-DHF-treated group. In brain cortex, the expression of AEP and tau N368 were also assessed (**Figures 3H,I**). Active-AEP was remarkably expressed at high levels in ApoE-KO group compared with both normal group (*p* < 0.01) and 7,8-DHF-treated group (*p* < 0.01). But lower expression of active-AEP was found in normal group than in 7,8-DHF-treated group (*P* < 0.01). The lowest expression of tau N368 was found in normal group (*p* < 0.05), but no significant difference was found between ApoE-KO group and 7,8-DHF-treated group. Taken together, the results indicated that consistent with the result of behavioral test, 7,8-DHF might play a role in ameliorating cognitive impairment by decreasing expression of active-AEP and its following truncated tau N368 of hippocampus. But in cortex, although with dramatic decrease in expression of AEP, 7,8-DHF failed to down-regulated expression of tau N368. It is noteworthy that both in cortex and hippocampus, ApoE-KO mice with high-fat diet expressed dramatic high levels in active-AEP and tau N368 that matched its poor cognitive condition. These results indicated that, the cognitive impairment of ApoE-KO might be associated with tau phosphorylation and truncation and the abnormal pathology of tau could be reduced by 7,8-DHF-induced neuroprotection via inhibiting activation of GSK-3β and AEP.

## Discussion

In our present study, we demonstrated that memory-deteriorating of 30-weeks old ApoE-KO mice with western type diet might be associated with higher expression of truncated tau N368 as well as active-AEP in both brain cortex and hippocampus compared with C57BL/6 mice at the same age, whereas, the phosphorylated tau S396 might not play a vital role in impairing cognitive function. We found that cognitive impairment could be reversed with long-term administration of 7,8-DHF, a TrkB receptor agonist, which could activate AKT/GSK3β signaling pathway as well as suppress expression of active-AEP, and then display its remarkable protective effects by decreasing production of tau N368 and phosphorylated tau at serine 396.

The underlying mechanisms of cerebrovascular disease risk factors, like atherosclerosis and hyperlipidemia, affecting cognition are poorly understood. ApoE-KO mice fed with high cholesterol diet resulted in severe atherosclerosis, increased leakage of blood-brain barrier, neuronal apoptosis, and cognitive impairment (Bink et al., 2013). Nowadays, vascular dysfunction seems to be closely related with AD-like pathological changes composed of Aβ and tau, the main components of plaques and tangles, respectively (Di Marco et al., 2015). ApoE has three isoforms-ε2 allele, ε3 allele, and ε4 allele, which plays an important role in nervous system via modulating Aβ metabolism. Many researches have reported the relationship between pathology of AD and ApoE genotype (DeMattos et al., 2004; Jiang et al., 2008; Kim et al., 2009; Morris et al., 2010; Liu et al., 2013; Poirier et al., 2014). The ApoE4 isoform, which is regarded as genetic risk factor of AD, exhibited poor ability in regulating Aβ clearance, therefore Aβ aggregation accelerated in the brain which boosted age-dependent cognitive impairment while the other two isoforms promoted effective removal of Aβ peptides from brain and reduced the risk of cognitive decline (Jiang et al., 2008; Liu et al., 2013). ApoE efficiently stimulates degradation of Aβ within the brain (Jiang et al., 2008). In addition to that, scientists have proved the increasing accumulation and deposition of Aβ in the absence of ApoE *in vivo* (DeMattos et al., 2004) and *in vitro* (Jiang et al., 2008), which were in line with cognitive impairment of this kind of mouse. Furthermore, potential AD-like mechanisms such as tau pathology, which might contribute to cognitive deficits in ApoE-KO mice (Oitzl et al., 1997), has not been ruled out (Bi et al., 2001). Our behavioral examination proved that 30-weeks old ApoE-KO mouse with western type diet for 25 weeks exhibited poor spatial learning and memory compared with C57BL/6 mouse with the same age and same diet. Consistent with poor performance of ApoE-KO mouse in MWM tests, AEP, which might be activate by acid PH, and its downstream truncated tau N368 were significantly increased in both cortex and hippocampus which indicated that tau N368 may mediate cognitive dysfunction in ApoE deficiency mouse. Interestingly, although some researches have proved expression of phosphorylated tau in ApoE-KO mouse, in our study, no significant difference was found in tau S396 between ApoE-KO mouse and C57BL/6 mouse, which suggested that tau S396 might not play a major role in alteration of cognition of ApoE-KO mouse at age of 30 weeks. Because antibody PHF1 have higher affinity in detecting tau peptides containing phosphorylated Ser396 and Ser404 than other tau pathology, it is understandable both ApoE-KO group and C57BL/6 group express similarly in PHF1.

Scientists have proved that administration of 7,8-DHF as a potent way to prevent or even rescue cognitive impairment in various diseases via multiple neuroprotective molecular mechanisms in both vivo and vitro. It preserved integration of synapses and synaptic plasticity (Zeng et al., 2012), protects neurons from toxicity of β-amyloidogenesis as well as reduces expression of BACE1 in five familial AD mutation mouse model of AD (Devi and Ohno, 2012). It could also ameliorate spatial memory deficits via increasing synapse protein level of AMPA subunits even in the absence of attenuating expression of amyloid precursor protein or Aβ in the Tg2576 AD mouse model (Gao et al., 2016). Besides AD-associated memory impairment, 7,8-DHF could also reverse memory deficits in schizophrenia (Yang et al., 2014), Fragile x syndrome (Tian et al., 2015), scopolamine-induced memory dysfunction (Chen et al., 2014), and age-related cognitive impairment (Zeng et al., 2012). But whether it could ameliorate cognitive deficits in ApoE-KO mice was still unknown. We have proved that cognitive decline of ApoE-KO mice might be obviously ameliorated with long-term administration of 7,8-DHF. We demonstrated that chronic treatment with 7,8-DHF significantly diminished expression of tau S396, PHF1, and tau N368 by inhibiting activation of GSK3β and reducing expression of active-AEP alternatively.

In Summary, our study indicated that cognitive deficit of ApoE-KO mice with western type diet might associated with truncated tau N368 due to the activation of AEP. Long-term treatment of 7,8-DHF could ameliorate cognitive impairment by inhibiting expression of tau pathology, which suggested a potential therapeutic strategy for 7,8-DHF in treating tau-related cognitive impairment. But how did 7,8-DHF acted on the activation of AEP deserve further study.

## Author Contributions

YT, SN, and WZ: participated in the design of the study, performed the experiments, analyzed the data, and drafted the manuscript. YZ and YL: conceived and designed the experiments, read and approved the final manuscript. FL, HG, JC, XC, and XJ: contributed reagents/materials/analysis tools. All authors read and approved the final manuscript.

## Conflict of Interest Statement

The authors declare that the research was conducted in the absence of any commercial or financial relationships that could be construed as a potential conflict of interest.
